# Ocular inflammatory events following COVID-19 vaccination: a multinational case series

**DOI:** 10.1186/s12348-021-00275-x

**Published:** 2022-01-04

**Authors:** Ilaria Testi, Camilo Brandão-de-Resende, Rupesh Agrawal, Carlos Pavesio, for the COVID-19 Vaccination Ocular Inflammatory Events Study Group, Laura Steeples, Balini Balasubramaniam, Peter McCluskey, Francesco Pichi, Aniruddha Agarwal, Carl Herbort, Luca Cimino, Salam Iriqat, Jennifer E. Thorne, Jose Echegaray, Kalpana Babu, Alexander Arthur Bialasiewicz, Debra A. Goldstein, Nima Ghadiri, Alex Fonollosa Calduch, Gabriel Costa de Andrade, Padmamalini Mahendradas, Julio J. González-López, Ester Carreño, Rola N. Hamam, Nicole Stübiger, Bahram Bodaghi, Yu-Jang Chao, Masaru Takeuchi, Mei-Ling Tay-Kearney, Alejandro Portero, Hiroshi Keino, Mar Esteban-Ortega, Joanna Przeździecka-Dołyk, Aleksandra Radosavljević, Ian Paredes, Rupesh Agrawal, Ho Su Ling, Wei Kiong, Melissa Tien, Xin Le Ng, Carlos Pavesio, Ilaria Testi, Soon Phaik Chee, Jay Siak, Ines Hernanz-Rodriguez, Victor Menezo, Christoph Tappeiner, Franz Marie Cruz, Peter Addison, Robert Kuijpers, Daniel Vitor Vasconcelos-Santos

**Affiliations:** 1 Department of Uveitis, Moorfields Eye Hospital, NHS Foundation Trust, London, UK; 2 Clinical Research Facility, Moorfields Eye Hospital, NHS Foundation Trust, London, UK; 3 National Healthcare Group Eye Institute, Tan Tock Seng Hospital, Singapore, Singapore; 4 Singapore Eye Research Institute, Singapore, Singapore; 5 Lee Kong Chian School of Medicine, Nanyang Technological University, Singapore, Singapore; 6 The Ophthalmology & Visual Sciences Academic Clinical Programme, Duke NUS Medical School, Singapore, Singapore

**Keywords:** Uveitis, Ocular inflammation, Immunomodulatory, Coronavirus disease, COVID-19, SARS-CoV-2, Vaccination

## Abstract

**Background:**

Inflammatory adverse events following COVID-19 vaccination are being reported amidst the growing concerns regarding vaccine’s immunogenicity and safety, especially in patients with pre-existing inflammatory conditions.

**Methods:**

Multinational case series of patients diagnosed with an ocular inflammatory event within 14 days following COVID-19 vaccination collected from 40 centres over a 3 month period in 2021.

**Results:**

Seventy patients presented with ocular inflammatory events within 14 days following COVID-19 vaccination. The mean age was 51 years (range, 19–84 years). The most common events were anterior uveitis (*n* = 41, 58.6%), followed by posterior uveitis (*n* = 9, 12.9%) and scleritis (*n* = 7, 10.0%). The mean time to event was 5 days and 6 days (range, 1–14 days) after the first and second dose of vaccine, respectively. Among all patients, 36 (54.1%) had a previous history of ocular inflammatory event. Most patients (*n* = 48, 68.6%) were managed with topical corticosteroids. Final vision was not affected in 65 (92.9%), whereas 2 (2.9%) and 3 (4.3%) had reduction in visual acuity reduced by ≤3 lines and > 3 lines, respectively. Reported complications included nummular corneal lesions (*n* = 1, 1.4%), cystoid macular oedema (*n* = 2, 2.9%) and macular scarring (*n* = 2, 2.9%).

**Conclusion:**

Ocular inflammatory events may occur after COVID-19 vaccination. The findings are based on a temporal association that does not prove causality. Even in the possibility of a causal association, most of the events were mild and had a good visual outcome.

The most common ocular manifestation of COVID-19, the disease cause by SARS-CoV-2, includes conjunctivitis, with reported cases of keratitis, keratoconjunctivitis, episcleritis, uveitis, posterior ischemic optic neuropathy and retinal vascular involvement [1–4]. In December 2020, the Food and Drug Administration (FDA) released the emergency use authorisation for the Pfizer-BioNTech and Moderna COVID-19 vaccine for the prevention of 2019 coronavirus disease, whereas the Oxford-AstraZeneca COVID-19 vaccine was authorised by the European Medicines Agency (EMA) soon after in January 2021. Currently, there are four types of COVID-19 vaccines available, including the messenger RNA (mRNA) vaccines (Pfizer-BioNTech and Moderna); the protein subunit vaccines (Novavax); the vector vaccines (Janssen Johnson & Johnson and Oxford-AstraZeneca), and the whole virus vaccines (Sinovac13, Sinopharm14 and Covaxin). Inflammatory adverse events, including myocarditis and pericarditis, have been reported to occur following COVID-19 vaccination [5, 6]. The objective of this study is to describe the spectrum and outcome of ocular inflammatory events associated with the administration of COVID-19 vaccination.

## Methods

Case series of patients diagnosed with an ocular inflammatory event after receiving COVID-19 vaccination collected from 40 international centres over a 3 month period in 2021 (see group information). The study was conducted with ethical approval obtained by the leading centre from its local institutional ethics committee (ethics approval: 2021/00438). The diagnosis of COVID-19 vaccination related-ocular inflammation was established based on the onset of the event within 14 days following COVID-19 vaccination and the patients who satisfied this specific criteria were only recruited for this report. A form for data collection was sent to all International Ocular Inflammation Society (IOIS) and International Uveitis Study Group (IUSG) members. Clinicians who observed an ocular inflammatory event within 14 days following COVID-19 vaccination filled out the form. The following information were retrieved from patients’ medical records: type of COVID-19 vaccination, timing of the events, including date of vaccination and date of onset of uveitis, past ocular history, type of ocular inflammatory event, including scleritis, episcleritis, anterior uveitis, posterior uveitis, intermediate uveitis, panuveitis, and optic neuritis, local and systemic treatment history, therapeutic management and outcome.

### Data collection

A purpose built data entry platform was created to collect the ocular inflammatory adverse reactions to COVID-19 vaccination. The secure encrypted web-based platform was programmed by CP, RA and IT. Given the observational and retrospective nature of the data, multiple imputations were not allowed. Ninety-four cases were collected, however, 24 had the event, 2 weeks after the vaccination and were excluded from the analysis, resulting in 70 cases. Statistical analysis was done using the software R v 4.1.1 [R Core Team (2021). R: A language and environment for statistical computing. R Foundation for Statistical Computing, Vienna, Austria]. Continuous variables were described as median [range] while binary variables were described as number (%).

## Results

Seventy patients presented with ocular inflammatory events within 14 days following COVID-19 vaccination, and the most common were anterior uveitis (*n* = 41, 58.6%), posterior uveitis (*n* = 9, 12.9%), and anterior scleritis (*n* = 7, 10.0%). The study population and the corresponding phenotypes is described in Table 1 with the details of ocular inflammatory events in Table 2.

**Table 1:** Description of the study population by the type of event (*n* = 70)

	Variable	Total (%)	Anterior Uveitis (%)	Posterior Uveitis (%)	Scleritis (%)	Others (%)
**Demographics**	**Number of Patients**	70 (100.0)	41 (58.6)	9 (12.9)	7 (10.0)	13 (18.6)
	^*^ **Age (years)**	51 [19–84]	55 [19–84]	40 [28–61]	48 [40–52]	54 [25–79]
	^*^ **Gender = Female**	35 (56.5)	19 (52.8)	4 (44.4)	6 (85.7)	6 (60)
	^*^ **Gender = Male**	27 (43.5)	17 (47.2)	5 (55.6)	1 (14.3)	4 (40)
	**History of Previous COVID-19**	1 (1.4)	1 (2.4)	0 (0.0)	0 (0.0)	0 (0.0)
**Vaccine**	**Pfizer**	40 (57.1)	20 (48.8)	4 (44.4)	5 (71.4)	11 (84.6)
	**Astra-Zeneca**	17 (24.3)	12 (29.3)	4 (44.4)	1 (14.3)	0 (0)
	**Moderna**	10 (14.3)	7 (17.1)	1 (11.1)	1 (14.3)	1 (7.7)
	**Sinopharm**	2 (2.9)	1 (2.4)	0 (0)	0 (0.0)	1 (7.7)
	**Covaxin**	1 (1.4)	1 (2.4)	0 (0)	0 (0.0)	0 (0)
**Event After First Dose**	**Number of Patients**	43 (61.4)	22 (53.7)	6 (66.7)	4 (57.1)	11 (84.6)
	**Time After First Dose [days]**	6 [1–14]	5.5 [1–14]	6.5 [1–14]	4.5 [1–9]	8 [1–14]
	**Received Another Dose**	18 (41.9)	11 (50)	3 (50)	1 (25)	3 (27.3)
	**Recurrence After Second Dose**	6 / 18 (33.3)	5 / 11 (45.5)	0 (0.0)	1 / 4 (25.0)	0 (0.0)
**Event After Second Dose**	**Number**	27 (39.6)	19 (46.3)	3 (33.3)	3 (42.9)	2 (15.4)
	**Time After Second Dose [days]**	5 [1–14]	5 [1–14]	8 [2–9]	4 [2–14]	7 [2–12]

^*^Missing Data for Age and Gender: Total = 8, Anterior Uveitis = 5, Others = 3

**Table 2:** Ocular Inflammatory Events Description

	Variable	Total (%) (N = 70)	Anterior Uveitis (%) (N = 41)	Posterior Uveitis (%) (N = 9)	Scleritis (%) (***N*** = 7)	Others (%) (N = 13)
**Previous Ocular Inflammatory Events**	**Number of patients**	36 (51.4)	23 (56.1)	3 (33.3)	5 (71.4)	5 (38.5)
	**Controlled more than 3 months**	28 (82.4)	17 (81.0)	3 (100)	3 (60.0)	5 (100)
	**On Topical Anti-inflammatory**	1 (2.8)	1 (4.3)	0 (0.0)	0 (0.0)	0 (0.0)
	**On Systemic Anti-inflammatory**	7 (19.4)	4 (17.4)	0 (0.0)	1 (20.0)	2 (15.4)
	**Event similar to previous events**	34 (94.4)	23 (100.0)	2 (66.7)	5 (100.0)	4 (80)
**Presentation**	**Unilateral**	60 (85.7)	33 (80.5)	8 (88.9)	7 (100)	12 (92.3)
	**Bilateral**	10 (14.3)	8 (19.5)	1 (11.1)	0 (0.0)	1 (7.7)
	**VA Unaffected**	38 (54.3)	22 (53.7)	2 (22.2)	6 (85.7)	8 (61.5)
	**VA reduced ≤ 3 lines**	18 (25.7)	10 (24.4)	4 (44.4)	1 (14.3)	3 (23.1)
	**VA reduced > 3 lines**	14 (20.0)	9 (22.0)	3 (33.3)	0 (0.0)	2 (15.4)
**Management**	**Topical Corticosteroids**	48 (68.6)	34 (82.9)	3 (33.3)	6 (85.7)	5 (38.5)
	**Systemic Corticosteroids**	13 (18.6)	2 (4.9)	6 (66.7)	1 (14.3)	4 (30.8)
	**Antivirals**	6 (8.6)	5 (12.2)	0 (0.0)	0 (0.0)	1 (7.7)
	**NSAIDs**	2 (2.9)	0 (0.0)	0 (0.0)	1 (14.3)	1 (7.7)
	**Antibiotics**	4 (5.7)	0 (0.0)	4 (44.4)	0 (0.0)	0 (0.0)
**Visual Outcomes**	**VA Unaffected**	65 (92.9)	39 (95.1)	7 (77.8)	7 (100)	12 (92.3)
	**VA reduced ≤ 3 lines**	2 (2.9)	1 (2.4)	0 (0.0)	0 (0.0)	1 (7.7)
	**VA reduced > 3 lines**	3 (4.3)	1 (2.4)	2 (22.2)	0 (0.0)	0 (0.0)
**Complications**	**Transient IOP elevation**	3 (4.3)	3 (7.3)	0 (0.0)	0 (0.0)	0 (0.0)
	**Nummular Corneal Lesions**	1 (1.4)	1 (2.4)	0 (0.0)	0 (0.0)	0 (0.0)
	**Cystoid Macular Oedema**	2 (2.9)	1 (2.4)	0 (0.0)	0 (0.0)	1 (7.7)
	**Macular Scarring**	2 (2.9)	0 (0.0)	2 (22.2)	0 (0.0)	0 (0.0)

Forty-one patients had anterior uveitis after vaccination, of which nine (22.0%) had history of HLA-B27 associated uveitis (one was on secukinumab and one on infliximab plus methotrexate), six (14.6%) had idiopathic anterior uveitis, three (7.3%) had glaucomatocyclitic crisis (one on topical corticosteroids), two (4.9%) with herpetic anterior uveitis, one (2.4%) of juvenile idiopathic arthritis associated uveitis (on adalimumab), one (2.4%) of Cytomegalovirus (CMV) uveitis and one (2.4%) of systemic lupus erythematosus uveitis (on hydroxychloroquine). All the events after vaccination in these patients were similar to their previous ones. Two patients (4.9%) presented with herpetic anterior uveitis for the first time and no other new diagnosis was made for any other patient. Two patients (4.9%) had persistent visual loss on the last follow-up, one (2.4%) due to cystoid macular oedema (visual acuity (VA) reduced ≤3 lines) and one (2.4%) due to nummular corneal lesions (VA reduced > 3 lines).

Nine patients had posterior uveitis after vaccination, of which 2 (22.2%) had history of ocular toxoplasmosis, and one (11.1%) of acute zonal occult outer retinopathy (AZOOR). The patients with history of ocular toxoplasmosis presented with recurrence of lesions and the patient with AZOOR had a different presentation from previous events, with multifocal choroiditis. Three patients (33.3%) presented with ocular toxoplasmosis, two (22.2%) presented with retinal vasculitis, and one (11.1%) presented with choroiditis for the first time. One patient (11.1%) with ocular toxoplasmosis, and one (11.1%) with occlusive retinal vasculitis had persistent visual loss on the last follow-up due to macular scarring.

Seven patients had anterior scleritis after vaccination, of which four (57.1%) had history of idiopathic anterior scleritis, and one (14.3%) of idiopathic posterior scleritis (on methotrexate and prednisolone). All the events after vaccination in these patients were similar to their previous ones. Two patients (28.6%) presented anterior scleritis for the first time. All patients had unaffected visual acuity after the event.

Other events (*N* = 13, 18.6%) included panuveitis (*N* = 3, 4.3%), optic neuritis (*N* = 2, 2.9%), episcleritis (N = 2, 2.9%), intermediate uveitis (N = 2, 2.9%), paracentral acute middle maculopathy (N = 1, 1.4%), giant cell arteritis (N = 1, 1.4%), periocular skin herpes zoster (N = 1, 1.4%), and unspecific blurriness of vision (N = 1, 1.4%). Among these patients, one (7.7%) had history of intermediate uveitis (was on mycophenolic acid), one (7.7%) of idiopathic panuveitis (was on azathioprine), one (7.7%) of optic neuritis, one (7.7%) of CMV anterior uveitis, and one (7.7%) of episcleritis. The events after vaccination in these patients were similar to their previous ones, except for the patient with history of CMV anterior uveitis, who presented with intermediate uveitis after vaccination. One patient (7.7%) had persistent visual loss on the last follow-up due to cystoid macular oedema in intermediate uveitis (VA reduced ≤3 lines).

Regarding the management of the events, 39 patients (55.7%) received only topical corticosteroids, 13 (18.6%) received systemic corticosteroids (five of them also received topical corticosteroids and four antibiotics), 10 (14.3%) did not require any treatment, six (8.6%) received antivirals (two of them with topical corticosteroids), and two (2.8%) received topical corticosteroids with oral non-steroidal anti-inflammatory drugs (NSAIDs). Three patients (4.3%) required IOP lowering medications (two with glaucomatocyclitic crisis and one with CMV anterior uveitis) and one (1.4%) initiated methotrexate after the episode (giant cell arteritis).

## Discussion

Although rare, development of uveitis after administration of vaccine is a known event. Cases of vaccine-associated uveitis have been reported with almost all the vaccines currently administered, including vaccines against hepatitis A and B virus, human papillomavirus, influenza virus, bacillus Calmette-Guerin, measles-mumps-rubella, varicella virus, yellow fever and Neisseria meningitides [7–18].

Ocular inflammatory events after COVID-19 vaccination from a multinational case series are described in this study. A search of Medline, using PubMed and Google Scholar, performed in August 2021, using the following keywords: ‘uveitis’, ‘vaccination’, ‘COVID-19’, revealed six case reports and two national case series describing presumed COVID-19 vaccine-related uveitis have been reported so far, including new onset of bilateral juvenile idiopathic arthritis (JIA)-associated anterior uveitis, unilateral anterior uveitis, bilateral choroiditis complicated by subretinal fluid, bilateral panuveitis with choroidal thickening and vascular leakage, and recurrence of Vogt Koyanagi Harada (VKH) [19–26]. Rabinovitch et al. reported twenty one cases of uveitis after COVID-19 vaccination in Israel, of which nineteen were diagnosed with anterior uveitis and two developed multiple evanescent white dot syndrome (MEWDS) [24]. Recently, Pichi et al. described seven patients diagnosed with episcleritis (1), anterior scleritis (2), acute macular neuroretinopathy (AMN) (2), paracentral acute middle maculopathy (PAMM) (1), and subretinal fluid (1) after COVID-19 vaccination [25]. None of these studies was a multicentral case series. Similarly to the findings of Rabinovitch et al., we found that anterior uveitis was the most common ocular inflammatory event observed after COVID-19 vaccination, with more than 50% of the patients having a known history of uveitis. The majority of the episodes we reported occurred after the Pfizer vaccine. These findings are probably based on the number of the administered doses.

The potential mechanism underlying the ocular inflammation response following COVID-19 vaccination is not known. Commonly proposed mechanisms include (1) molecular mimicry secondary to resemblance between uveal peptides and vaccine peptide fragments, (2) antigen-specific cell and antibody-mediated hypersensitivity reactions, (3) inflammatory damage induced by adjuvants included the vaccines stimulating innate immunity through endosolic or cytoplasmic nucleic acid receptors [18, 27–30].

The Bradford-Hill criteria include nine aspects to consider when inferring causality between events: (1) strength of the association, (2) consistency, (3) specificity, (4) temporality, (5) biological gradient, (6) plausibility, (7) coherence, (8) experiment, and analogy (9) [31]. The design of this study guarantees temporality, since there was a short interval of time following vaccination and the onset of the events. The fact that among 18 patients who had an event following the first dose and still received a second dose, 6 (33.3%) presented similar events following the second dose, as well as different observations in different regions from different people, supports consistency (reproducibility) between exposure and outcome. The causality is plausible as some biological mechanisms have already been proposed. Finally, the events after COVID-19 vaccination are analogous to those reported following other vaccines.

From our study it emerged that most of the inflammatory episodes were not severe, and patients were mainly managed with topical corticosteroids or observation only (70%), with unaffected final visual acuity (92.9%). Very few patients developed ocular complications resulting in tissue damage and visual loss (7.1%), including corneal opacity, cystoid macular oedema and macular scarring.

Study limitations include its observational and non-controlled nature, that cannot be used to infer causality because it does not allow to stablish strength of the association, specificity, biological gradient, coherence, nor experiment. Additionally, there is a potential sample bias due to the form of data collection, and the data collected might not be representative of all population, although the multicentre nature of the study need to be considered as it may reduce the bias.

## Conclusion

Hereby we report a large multinational case series of ocular inflammatory events occurred following COVID-19 vaccination, based on a temporal and multicentrical association, but not proving causality. Even in the possibility of of a causal association, most of the events were mild and had a good visual outcome. Therefore, there is no evidence from this study to suggest that individuals should avoid getting vaccinated because of ophthalmic-related adverse events. It is not the aim of the authors to impede or curtail the vaccination efforts, but rather to educate physicians and patients about rare but potential ocular inflammatory events after the COVID-19 vaccination.

## Data Availability

All the data pertaining to the cases are available with the corresponding author.

## Abbreviations

FDA: Food and drug administration
EMA: European medicines agency
mRNA: Messenger RNA
AZOOR: Acute zonal occult outer retinopathy
VA: Visual acuity
CMV: Cytomegalovirus
NSAIDs: Non-steroidal anti-inflammatory drugs
JIA: Juvenile idiopathic arthritis
VKH: Vogt Koyanagi Harada
MEWDS: Multiple evanescent white dot syndrome
AMN: Acute macular neuroretinopathy
PAMM: Paracentral acute middle maculopathy
